# mtDNA variability determines spontaneous joint aging damage in a conplastic mouse model

**DOI:** 10.18632/aging.204153

**Published:** 2022-07-02

**Authors:** Morena Scotece, Carlos Vaamonde-García, Ana Victoria Lechuga-Vieco, Alberto Centeno Cortés, María Concepción Jiménez Gómez, Purificación Filgueira-Fernández, Ignacio Rego-Pérez, José Antonio Enríquez, Francisco J. Blanco

**Affiliations:** 1 Unidad de Genómica, Grupo de Investigación de Reumatología (GIR), Instituto de Investigación Biomédica de A Coruña (INIBIC), Complexo Hospitalario Universitario de A Coruña (CHUAC), Sergas, Universidade da Coruña (UDC), A Coruña 15006, Spain; 2 Universidade da Coruña (UDC), Grupo de Investigación de Reumatología y Salud (GIR-S), Departamento de Biología, Campus de Zapateria, A Coruña 15011, Spain; 3 Centro Nacional de Investigaciones Cardiovasculares Carlos III, Madrid 28029, Spain; 4 CIBERES: C/ Melchor Fernández-Almagro 3, Madrid 28029, Spain; 5 Kennedy Institute of Rheumatology, University of Oxford, Headington, Oxford OX3 7FY, UK; 6 Centro Tecnológico de Formación Xerencia de Xestión Integrada A Coruña (XXIAC), A Coruña 15006, Spain; 7 Centro de Investigación Biomédica en Red de Bioingeniería, Biomateriales y Nanomedicina (CIBER-BBN), Madrid 28029, Spain; 8 Centro de Investigación Biomédica en Red de Fragilidad y Envejecimiento Saludable (CIBERFES), Instituto de Salud Carlos III, C/ Melchor Fernández-Almagro 3, Madrid 28029, Spain; 9 Universidade da Coruña (UDC), Grupo de Investigación de Reumatología y Salud (GIR-S), Departamento de Fisioterapia, Medicina y Ciencias Biomédicas, Facultad de Fisioterapia, Campus de Oza, A Coruña 15008, Spain

**Keywords:** conplastic mice, mtDNA, senescence, autophagy, oxidative stress

## Abstract

Mitochondria and mtDNA variations contribute to specific aspects of the aging process. Here, we aimed to investigate the influence of mtDNA variation on joint damage in a model of aging using conplastic mice. A conplastic (BL/6^NZB^) mouse strain was developed with the C57BL/6JOlaHsd nuclear genome and NZB/OlaHsd mtDNA, for comparison with the original C57BL/6JOlaHsd strain (BL/6^C57^). Conplastic (BL/6^NZB^) and BL/6^C57^ mice were sacrificed at 25, 75, and 90 weeks of age. Hind knee joints were processed for histological analysis and joint pathology graded using the Mankin scoring system. By immunohistochemistry, cartilage expression of markers of autophagy (LC3, Beclin-1, and P62) and markers of senescence (MMP13, beta-Galactosidase, and p16) and proliferation (Ki67) were analyzed. We also measured the expression of 8-oxo-dG and cleaved caspase-3.

Conplastic (BL/6^NZB^) mice presented lower Mankin scores at 25, 75, and 90 weeks of age, higher expression of LC3 and Beclin-1 and lower of P62 in cartilage than the original strain. Moreover, the downregulation of MMP13, beta-Galactosidase, and p16 was detected in cartilage from conplastic (BL/6^NZB^) mice, whereas higher Ki67 levels were detected in these mice. Finally, control BL/6^C57^ mice showed higher cartilage expression of 8-oxo-dG and cleaved caspase-3 than conplastic (BL/6^NZB^) mice. This study demonstrates that mtDNA genetic manipulation ameliorates joint aging damage in a conplastic mouse model, suggesting that mtDNA variability is a prognostic factor for aging-related osteoarthritis (OA) and that modulation of mitochondrial oxidative phosphorylation (OXPHOS) could be a novel therapeutic target for treating OA associated with aging.

## INTRODUCTION

Aging involves the progressive loss of physiological integrity, with consequent time-dependent functional decline and increased vulnerability to mortality. The aging phenotype is determined by several cellular and molecular hallmarks, such as genomic instability, cellular senescence, telomere attrition, and mitochondrial dysfunction [1].

Aging-related changes affect all of the joint tissues, including cartilage, menisci, subchondral bone, synovium, and ligaments. Among these tissues, the articular cartilage, with alterations in chondrocytes and the disruption of extracellular matrix homeostasis, presents the most intense aging-related changes. In association with other risk factors, such as obesity, mechanical injury, and genetics, these aging-related changes may lead to an earlier onset or the development of a more severe form of osteoarthritis (OA) [2]. However, it is important to note that OA is not an inevitable consequence of aging. Joint aging is a process that is distinct from the changes caused by the initiation of OA [3].

Many *in vitro* and *in vivo* studies have shown the contribution of mitochondria to various processes associated with aging, including inflammation, senescence, and age-dependent decline in tissue and organ function [4]. Interestingly, studies using transmitochondrial cybrids have demonstrated that mitochondria and mtDNA variation can regulate different nuclear target genes [5].

Increased production of ROS with consequent mtDNA damage is a result of dysfunction of the mitochondrial respiratory complex, which characterizes the aging phenotype of OA [6]. Apoptosis, decreased autophagy, and progressive telomere shortening accompany this gradual decline in mitochondrial function, which results in an increase of cartilage matrix destruction [7, 8]. Regarding the direct relationship between mitochondrial haplogroups and aging, several studies found that certain mtDNA variants, including the mtDNA haplogroup J, have a protective role against aging with a decrease in ROS production and a consequent decline in mtDNA damage [9, 10]. Moreover, many studies have reported the association of these mtDNA variants with increased human longevity [11–13]. Interestingly, in OA, haplogroup J was found to be associated with less apoptosis and the production of nitric oxide by articular chondrocytes [14]. However, such association studies are affected by various confounding factors making the assessment of patients difficult, such as nuclear genetic variability, lifestyle differences, and environmental factors.

An interesting tool that can be used to control for confounding effects is conplastic mice [13]. These mice have the same nuclear background, but different wild-type mtDNA variants, and are maintained under identical conditions. This allows study of the functions of the mtDNA variants in an *in vivo* context. Various strains of conplastic mice have been developed [14]. In a recent study, we showed that the extension of a healthy life was highly dependent on the mtDNA variant [15]. In the current study, the conplastic mice employed were generated with the C57BL/6JOlaHsd nuclear genome and NZB/OlaHsd mtDNA. The difference between the mtDNA of the NZB/B1NJ strain and BL/6J mtDNA resembles the sequence difference between human mtDNAs of distant populations, at around 90 single-nucleotide polymorphisms (SNPs). The mtDNAs of C57BL/6 and NZB/OlaHsd mice differ by 12 missense mutations, 4 transfer RNA (tRNA) mutations, 8 ribosomal RNA (rRNA) mutations, and 10 non-coding-region mutations. These correspond to a level of divergence comparable to that between human Eurasian and African mtDNAs [15].

In this study, we evaluated whether the substitution of the mtDNA variant from C57BL/6JOlaHsd with the mtDNA from NZB/OlaHsd would change the aging-related joint damage. For this purpose, we used an aging model to analyze the joint alterations in both strains. Using histological and immunohistochemical assays, we found that the mtDNA replacement reduced aging-related joint damage.

## RESULTS

### Aging process in joints from BL/6^C57^ and conplastic (BL/6^NZB^) mice

To analyze the influence of mtDNA variants on joint aging, we first performed safranin O staining of the right and left knee joints of mice at 25, 75, and 90 weeks of age in both conplastic (BL/6^NZB^) and BL/6^C57^ strains. The pathological changes in knee joints were quantified by a modified Mankin scoring system analyzing three criteria in the cartilage: structure, cellularity, and matrix staining [16].

**Figure 1:**
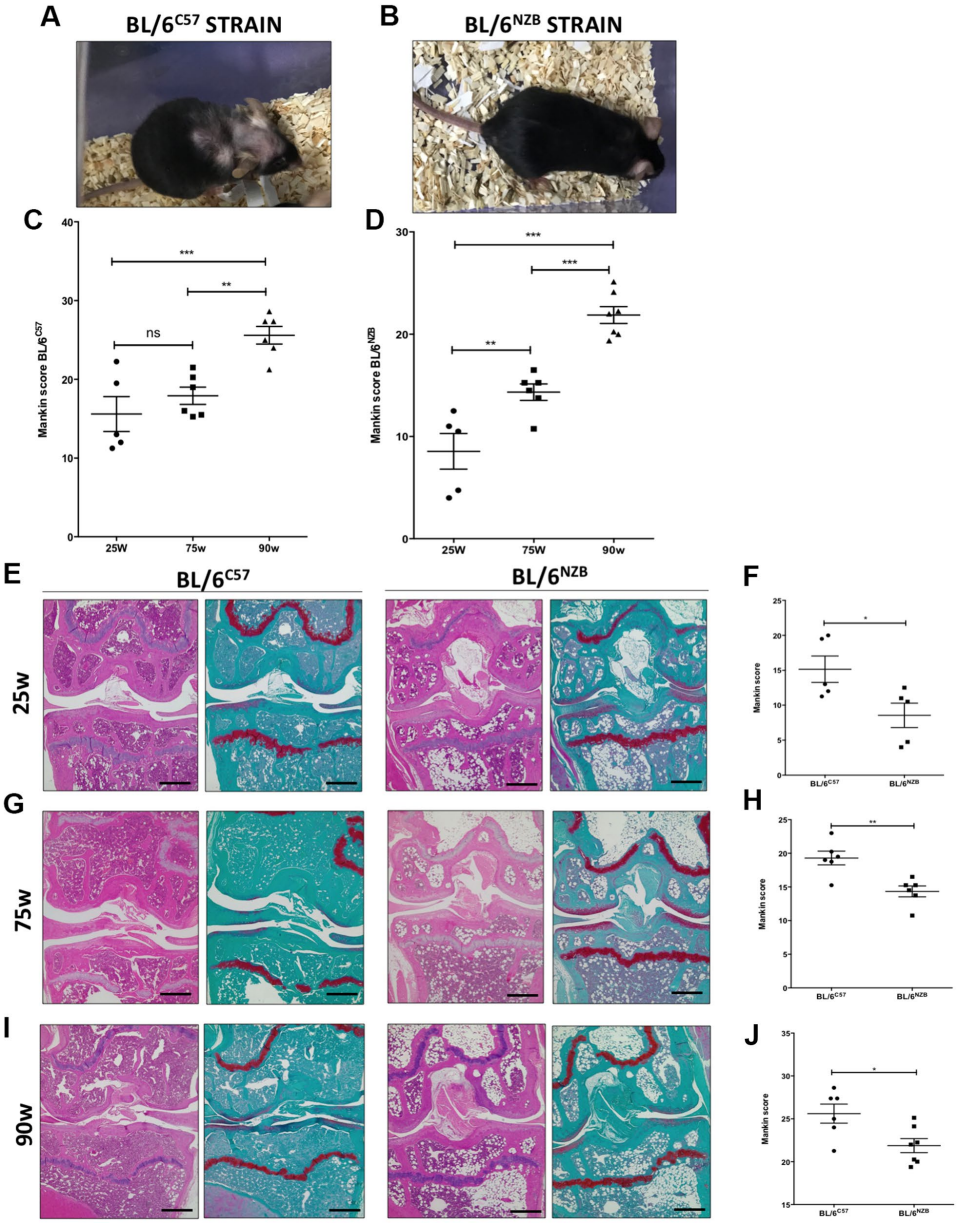
**Analysis of Mankin scores in joints from BL/6^C57^ and conplastic (BL/6^NZB^) mice during the aging process.** (**A**) Representative image of 90-weeks-old BL/6^C57^ mice. (**C**) Quantification of total Mankin score of the joint sections from BL/6^C57^ mice. (**B**) Representative image of 90-weeks-old conplastic (BL/6^NZB^) mice. (**D**) Quantification of total Mankin score of the joint sections from conplastic (BL/6^NZB^) mice. Total Mankin score was obtained from the scores of all quadrants of the joint (medial tibial plateau, medial femoral condyle, lateral tibial plateau, and lateral femoral condyle) that were scored separately and averaged. Three criteria were selected for histological assessment of each quadrant: structure, cellularity, and matrix staining. Graphs represent means ± SEM; n=5 in BL/6^C57^ and conplastic (BL/6^NZB^) mice at 25 weeks; n=6 in BL/6^C57^ and BL/6^NZB^ at 75 weeks; n=6 in BL/6^C57^ and n=7 in BL/6^NZB^ at 90 weeks. **p<0.01; ***p<0.001; ns=not significant by non-parametric Mann-Whitney test. (**E**) Representative images from joint sections of BL/6^C57^ and conplastic (BL/6^NZB^) mice at 25 weeks of age, stained with Hematoxylin-Eosin and Safranin O/Fast Green. (**F**) Quantification of total Mankin score of the joint sections from BL/6^C57^ vs. conplastic (BL/6^NZB^) mice at 25 weeks. (**G**) Representative images from joint sections of BL/6^C57^ and conplastic (BL/6^NZB^) mice at 75 weeks of age, stained with Hematoxylin-Eosin and Safranin O/Fast Green. (**H**) Quantification of total Mankin score of the joint sections from BL/6^C57^ vs. conplastic (BL/6^NZB^) mice at 75 weeks. (**I**) Representative images from joint sections of BL/6^C57^ and conplastic (BL/6^NZB^) mice at 90 weeks of age, stained with Hematoxylin-Eosin and Safranin O/Fast Green. (**J**) Quantification of total Mankin score of the joint sections from BL/6^C57^ vs. conplastic (BL/6^NZB^) mice at 90 weeks. Original magnification: 4×. Scale bar, 500 μm. Graphs represent means ± SEM; n=5 in BL/6^C57^ and conplastic (BL/6^NZB^) mice at 25 weeks; n=6 in BL/6^C57^ and conplastic (BL/6^NZB^) mice at 75 weeks; n=6 in BL/6^C57^ and n=7 in conplastic (BL/6^NZB^) mice at 90 weeks. *p<0.05; **p<0.01 vs. BL/6^C57^ by non-parametric Mann-Whitney test.

Increased cartilage Mankin score was observed in the original strain BL/6^C57^ (Figure 1A, 1C) and in the conplastic (BL/6^NZB^) mice (Figure 1B, 1D) at 25, 75, and 90 weeks of age, in line with the aging process. Moreover, mice from the conplastic (BL/6^NZB^) strain presented an increased Mankin score at 75 weeks when compared with mice at 25 weeks of age (Figure 1D, p=0.0087). However, we did not observe a significant difference in the Mankin scores of strain BL/6^C57^ between 25 and 75 weeks of age (Figure 1C, p=0.8782). In both strains, we showed strong and significant increases of the score between 25 and 90 weeks (Figure 1C, 1D, p<0.0001) and 75 and 90 weeks of age (Figure 1C, 1D, p=0.0058, p<0.0001).

We compared the development of the aging process at the joint level between the conplastic (BL/6^NZB^) mice and the original strain BL/6^C57^. In response to aging, compared with the BL/6^C57^ mice at the same age, the conplastic (BL/6^NZB^) mice presented significantly reduced Mankin scores at 25 (Figure 1E, 1F, p=0.0317), 75 (Figure 1G, 1H, p=0.0087), and 90 weeks of age (Figure 1I, 1J, p=0.0484).

All quadrants of the joint (medial tibial plateau, medial femoral condyle, lateral tibial plateau, and lateral femoral condyle) were scored separately and averaged (Supplementary Figure 1). Thereby, we showed reduced scores in both femoral condyle (FC) and tibial plateau (TP) of BL/6^NZB^ mice, which reached statistical significance at 75 weeks (FC: p=0.0089, TP: p=0.0155) and 90 weeks of age (FC: p=0.1906, TP: p=0.0466), when compared with the BL/6^C57^ mouse strain. In comparison with that in the BL/6^C57^ mouse strain, the Mankin score of the medial compartment (Med comp) was significantly reduced in the joints from conplastic (BL/6^NZB^) mice at all ages analyzed (25w: p=0.0397, 75w: p=0.0108, and 90w: p=0.0052) (Table 1). We observed significant decreases of Mankin score in the medial femoral condyle (MFC) at 25 (p=0.0317), 75 (p=0.0087), and 90 weeks of age (p=0.0251) (Table 1). Taken into account these results, the following analysis of the study were performed in medial compartment of the joint.

**Table 1:** Mankin score of the four joint quadrants in BL/6^C57^ vs. conplastic (BL/6^NZB^) mice at 25, 75, and 90 weeks of age.

**WEEKS**	**STRAIN**	**LTP**	**LFC**	**MTP**	**MFC**	**TP**	**FC**	**Med comp**	**Lat comp**	**Whole joint**
**25**	**BL/6^C57^**	3,30±0,64	3,95±0,67	3,40±0,70	4,95±0,55	6,70±1,21	8,90±1,09	8,35±1,06	7,25±1,28	15,15±1,90
	**BL/6^NZB^**	1,40±0,52	2,55±0,50	1,75±0,63	2,85±0,39*	3,15±0,97	5,40±0,83	4,60±0,95*	3,95±0,91	8,55±1,74*
**75**	**BL/6^C57^**	5,25±0,34	4,42±0,15	4,29±0,49	5,33±0,43	9,54±0,68	9,75±0,55	9,62±0,81	9,66±0,39	19,29±1,02
	**BL/6^NZB^**	3,58±0,44*	4,50±0,46	2,87±0,43	3,37±0,35**	6,45±0,51*	7,87±0,53*	6,25±0,61*	8,08±0,66	14,33±0,80**
**90**	**BL/6^C57^**	5,04±0,48	6,72±0,59	5,93±0,48	7,89±0,42	10,98±0,68	14,63±1,01	13,83±0,63	11,77±0,71	25,60±1,11
	**BL/6^NZB^**	4,42±0,29	6,57±0,40	4,42±0,33*	6,44±0,33*	8,85±0,29*	13,02±0,69	10,88±0,45**	11,00±0,52	21,88±0,81*

LTP, lateral tibial plateau; LFC, lateral femoral condyle; MTP, medial tibial plateau; MFC, medial femoral condyle; TP, tibial plateau=LTP+MTP; FC, femoral condyle=LFC+MFC; med comp, medial compartment=MTP+MFC; Lat comp, lateral compartment=LTP+LFC; whole joint=lateral compartment + medial compartment. Values represent means ± SEM; n=5 in BL/6^C57^ and conplastic (BL/6^NZB^) mice at 25 weeks; n=6 in BL/6^C57^ and conplastic (BL/6^NZB^) mice at 75 weeks; n=6 in BL/6^C57^ and n=7 in conplastic (BL/6^NZB^) mice at 90 weeks. *p<0.05, **p<0.01 vs. BL/6^C57^ by non-parametric Mann-Whitney test.

In addition, we evaluated the presence of pathological changes in other particular tissues, i.e. synovium and subchondral bone by semi-quantitative scoring systems and growth plate of tibial epiphysis by histomorphometry analysis (Supplementary Figure 2). However, no significant differences were detected between conplastic and original strain mice in term of pathological changes in the synovium and subchondral bone (Supplementary Figure 2A, 2B, 2D, 2E). Although a reduction of epiphysial plate was observed in BL/6^C57^ mice at 75 weeks of age compared with that in BL/6^NZB^ mice at the same age (Supplementary Figure 2C, 2F).

### Cartilage from conplastic (BL/6^NZB^) mice presented higher expression of autophagy markers than that from the original strain

**Figure 2:**
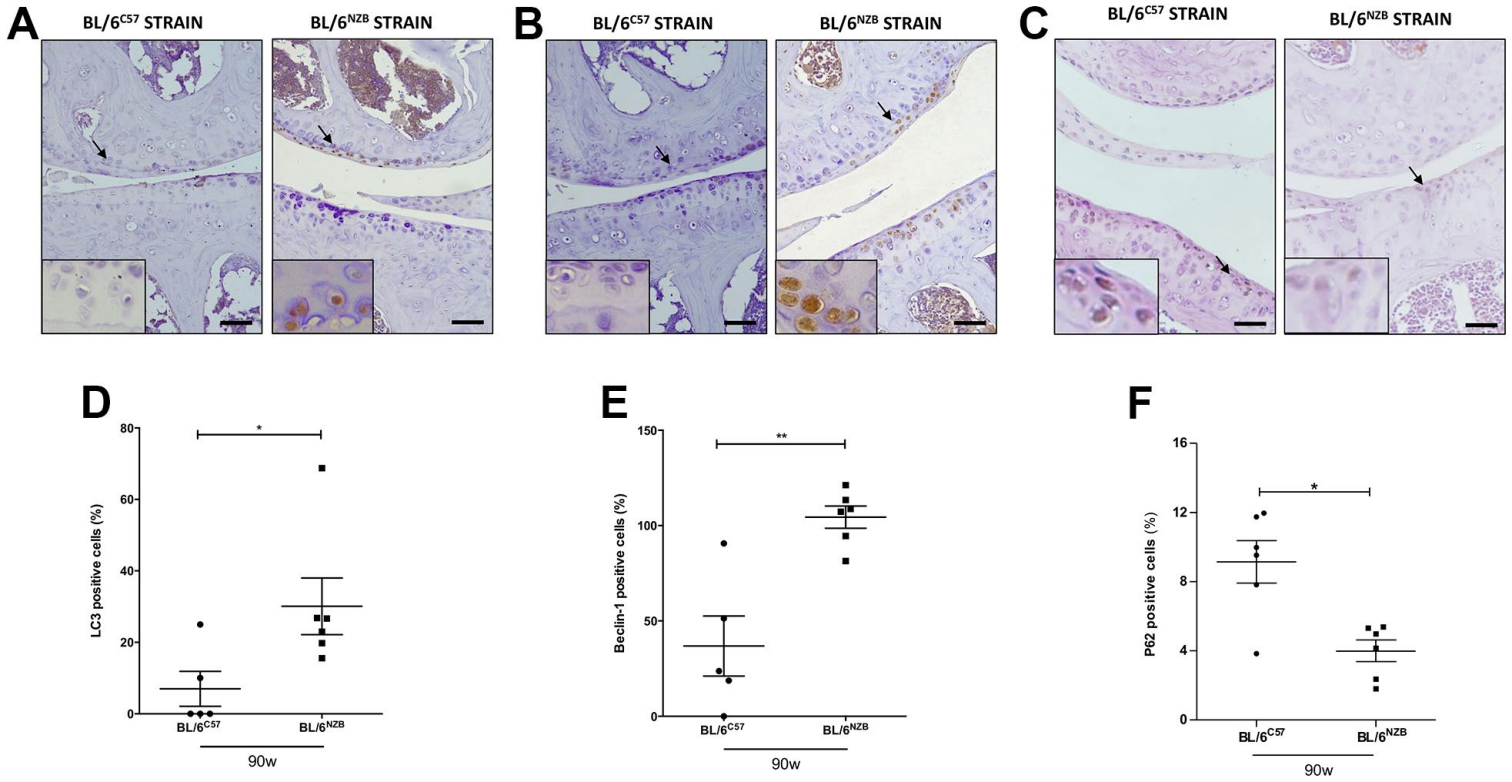
**Immunohistochemistry for markers of autophagy on mouse knee joint sections.** Representative images of medial compartment of knee joints from BL/6^C57^ and conplastic (BL/6^NZB^) mice at 90 weeks of age stained with LC3 (**A**), Beclin-1 (**B**) and P62 (**C**). Quantitative analysis of LC3-positive cells (**D**), Beclin-1-positive cells (**E**), and P62-positive cells (**F**) of knee joints from BL/6^C57^ and conplastic (BL/6^NZB^) mice at 90 weeks. Original magnification: 20×. Scale bar, 50 μm. Black arrow indicates positively stained chondrocyte. Chondrocyte magnification (40×) is shown in the bottom-left corner of the images. Graphs represent means ± SEM; n=5 in BL/6^C57^ and n=6 conplastic (BL/6^NZB^) mice for LC3 and Beclin-1, and n=6 in BL/6^C57^ and n=6 conplastic (BL/6^NZB^) mice for P62. *p<0.05; **p<0.01 by non-parametric Mann-Whitney test.

The autophagic capacity of cells declines naturally with age. To determine whether there were potential age-associated changes in autophagy-related proteins, we performed immunohistochemical analysis for LC3 and Beclin-1, two major regulators of the autophagy pathway, and P62, a classical receptor of autophagy that is degraded and in turn its protein levels reduced when the autophagy is activated. Cartilage expression of LC3 (Figure 2A, 2D, p=0.0281) and Beclin-1 (Figure 2C, 2F, p=0.0087) in the medial compartment of the joints was significantly reduced in the BL/6^C57^ mice at 90 weeks compared with that in the conplastic (BL/6^NZB^) mice at the same age. Whereas, P62 expression was significantly attenuated in BL/6^NZB^ mice compared with observed in wild type (BL/6^C57^) mice (Figure 2C, 2F, p=0.0260). We also observed these significant differences in LC3 (Supplementary Figure 3C, 3D) and Beclin-1 (Supplementary Figure 3G, 3H) expression at 75 weeks of age. However, we did not detect differences at 25 weeks of age (LC3: Supplementary Figure 3A, 3D; Beclin-1: Supplementary Figure 3E, 3F).

### Expression of senescence markers was delayed in cartilage from conplastic (BL/6^NZB^) mice

**Figure 3:**
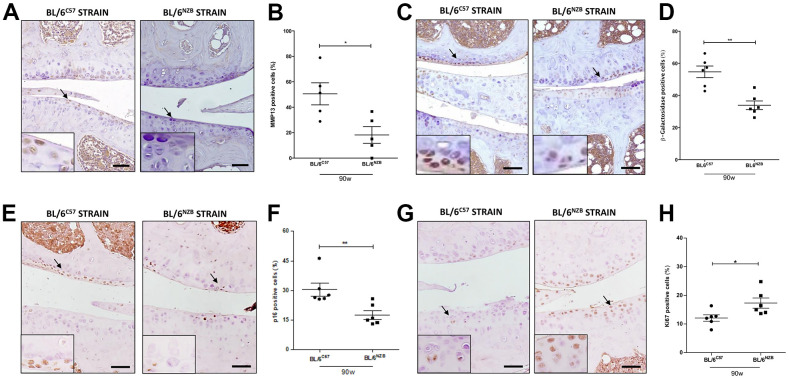
**Age-associated changes in cartilage expression of senescence and proliferation markers in BL/6^C57^ and conplastic (BL/6^NZB^) mice.** (**A**) Representative images of medial compartment of knee joints from BL/6^C57^ and conplastic (BL/6^NZB^) mice at 90 weeks of age stained with matrix metalloproteinase 13 (MMP13). (**B**) Quantitative analysis of MMP13-positive cells of knee joints from BL/6^C57^ and conplastic (BL/6^NZB^) mice at 90 weeks of age. (**C**) Representative images of medial compartment of knee joints from BL/6^C57^ and conplastic (BL/6^NZB^) mice at 90 weeks of age stained with β-Galactosidase. (**D**) Quantitative analysis of β-Galactosidase-positive cells of knee joints from BL/6^C57^ and conplastic (BL/6^NZB^) mice at 90 weeks of age. (**E**) Representative images of medial compartment of knee joints from BL/6^C57^ and conplastic (BL/6^NZB^) mice at 90 weeks of age stained with p16. (**F**) Quantitative analysis of p16-positive cells of knee joints from BL/6^C57^ and conplastic (BL/6^NZB^) mice at 90 weeks of age. (**G**) Representative images of medial compartment of knee joints from BL/6^C57^ and conplastic (BL/6^NZB^) mice at 90 weeks of age stained with Ki67. (**H**) Quantitative analysis of Ki67-positive cells of knee joints from BL/6^C57^ and conplastic (BL/6^NZB^) mice at 90 weeks of age. Original magnification: 20×. Scale bar, 50 μm. Black arrow indicates positively stained chondrocyte. Chondrocyte magnification (40×) is shown in the bottom-left corner of the images. Graphs represent means ± SEM; n=5 in BL/6^C57^ and n=5 in conplastic (BL/6^NZB^) mice at 90 weeks of age for MMP13, and n=6 in BL/6^C57^ and n=6 in conplastic (BL/6^NZB^) mice at 90 weeks of age for β-Galactosidase, p16, and Ki67. *p<0.05, * *p<0.01 by non-parametric Mann-Whitney test.

To evaluate the senescence process and the potential difference between the conplastic (BL/6^NZB^) mice and the original strain, we analyzed the cartilage expression of different markers of senescence, i.e., matrix metalloproteinase 13 (MMP13), β-Galactosidase, and p16 and proliferation, Ki67, by immunohistochemistry. The conplastic (BL/6^NZB^) mice showed the downregulated expression of both MMP13 (Figure 3A, 3B, p=0.0317) and β-Galactosidase (Figure 3C, 3D, p=0.0043), and p16 proteins (Figure 3E, 3F, p=0.0043) when compared with the original strain BL/6^C57^ at 90 weeks. Whereas the levels of Ki67 (Figure 3G, 3H, p=0.0152) was higher in conplastic (BL/6^NZB^) than in original strain mice BL/6^C57^ at 90w. Conversely, immunohistochemical analysis of MMP13 in cartilage tissue sections at 25 and 75 weeks did not show significant differences between the two strains (Supplementary Figure 4A–4D). Moreover, we observed a trend of a decrease in β-Galactosidase expression in cartilage from conplastic (BL/6^NZB^) mice at 75 weeks of age (Supplementary Figure 4E–4H).

### Cartilage from BL/6^C57^ mice showed more oxidative stress than cartilage from conplastic (BL/6^NZB^) mice

**Figure 4:**
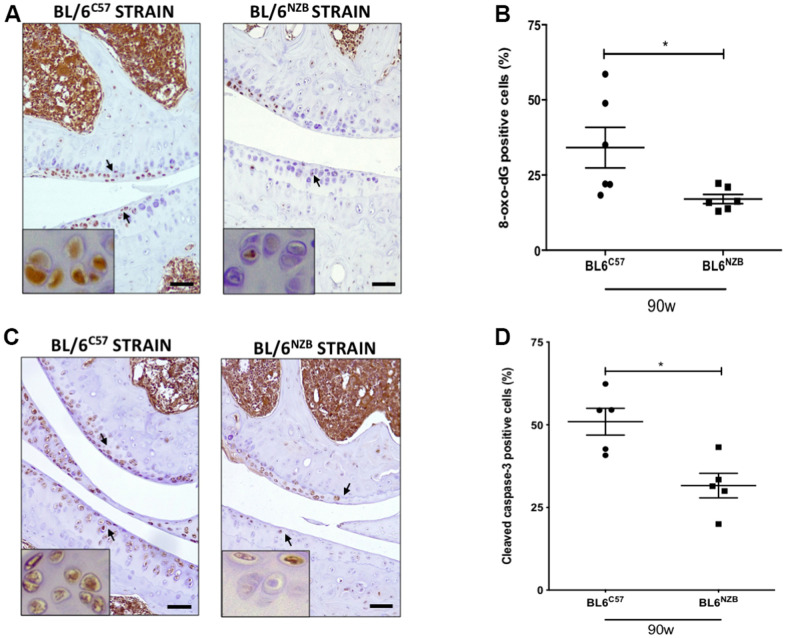
**Reduction of 8-oxo-dG and cleaved caspase-3 expression in cartilage from conplastic (BL/6^NZB^) mice.** (**A**) Representative images of medial compartment of knee joints from BL/6^C57^ and conplastic (BL/6^NZB^) mice at 90 weeks of age stained with 8-oxo-2′-deoxyguanosine (8-oxo-dG). (**B**) Quantitative analysis of 8-oxo-dG-positive cells of knee joints from BL/6^C57^ and conplastic (BL/6^NZB^) mice at 90 weeks. (**C**) Representative images of medial compartment of knee joints from BL/6^C57^ and conplastic (BL/6^NZB^) mice at 90 weeks of age stained with cleaved caspase-3. (**D**) Quantitative analysis of cleaved caspase-3-positive cells of knee joints from BL/6^C57^ and conplastic (BL/6^NZB^) mice at 90 weeks of age. Original magnification: 20×. Scale bar, 50 μm. Black arrow indicates positively stained chondrocyte. Chondrocyte magnification (40×) is shown in the bottom-left corner of the images. 8-oxo-dG: Graphs represent means ± SEM; n=6 in BL/6^C57^ and n=6 in conplastic (BL/6^NZB^) mice at 90 weeks of age. Cleaved caspase-3: Graphs represent means ± SEM; n=5 in BL/6^C57^ and n=5 in conplastic (BL/6^NZB^) mice at 90 weeks of age; *p<0.05 by non-parametric Mann-Whitney test.

We next investigated whether there was a difference in the expression of 8-oxo-dG, one of the major products of DNA oxidation [17, 18]. Cartilage expression of 8-oxo-dG was significantly downregulated in conplastic (BL/6^NZB^) mice at 90 weeks of age compared with that in the original strain (Figure 4A, 4B, p=0.0260). In addition, 8-oxo-dG expression was significantly reduced in the conplastic (BL/6^NZB^) mice also at 25 and 75 weeks of age (Supplementary Figure 5).

### Analysis of cleaved caspase-3 in cartilage from conplastic (BL/6^NZB^) mice and original strain

To evaluate apoptosis, we measured cleaved caspase-3, which is activated in apoptotic cells by both extrinsic and intrinsic pathways. In panels A and B, we show the reduction of cleaved caspase-3 in cartilage from conplastic (BL/6^NZB^) mice at 90 weeks of age when compared with that of the original strain (Figure 4C, 4D, p=0.0317). At 25 and 75 weeks of age, we observed slight decreases of cleaved caspase-3 expression in cartilage from the BL/6^NZB^ conplastic mice compared with that from the BL/6^C57^ mice at the same ages, but these did not reach statistical significance (Supplementary Figure 5).

## DISCUSSION

Aging is a biological process characterized by the disruption of cellular homeostasis, progressive tissue deterioration, and an increased risk of cell death [1]. As individuals age, their joints show some changes in mobility due in part to alterations in the connective tissues.

The cellular changes that occur during aging contribute to the development of OA, but OA is not a simple consequence of joint aging. In fact, not all older adults develop this pathology. Thus, OA is a multifactorial disease, of which aging is one of the major risk factors but not the only one. Indeed, there are important differences between an aged joint and one with OA. The link between aging and OA was believed to be only a result of continuous mechanical “wear and tear” of articular cartilage. However, the age-related changes to cartilage matrix and the progressive accumulation of damaged organelles and macromolecules are now known to contribute to the initiation and progression of cartilage degradation [19]. Several hallmarks of aging, including genomic instability, cellular senescence, epigenetic alterations, and mitochondrial dysfunction, contribute to OA [20].

The aging phenotype of OA is characterized by a change in the activity of mitochondrial respiratory complexes, which causes an increase of ROS production and consequent mtDNA damage [6]. Many *in vitro* studies have found an association between aging and mtDNA variants [9, 21–23]. For *in vivo* analysis of the functional role of mtDNA variants in aging-related joint damage, we generated for the first time to our knowledge a mouse model of spontaneous aging in conplastic mouse strains.

Conplastic mice have emerged as a potent tool to study the function of mtDNA variants in an *in vivo* setting. These mice present the same nuclear background, but have different mtDNA variants. Here, we used two wild-type mtDNA variants derived from C57BL/6JOlaHsd (BL/6^C57^) and NZB/OlaHsd (BL/6^NZB^), which correspond to a level of divergence comparable to that between human Eurasian and African mtDNAs. The proof-of-concept study demonstrated that the mtDNA haplotype profoundly influences mitochondrial proteostasis and reactive oxygen species generation, insulin signaling, and aging parameters, including telomere shortening and mitochondrial dysfunction, resulting in profound differences in health longevity between conplastic strains [15]. Notably, the abnormal accumulation of mtDNA mutations (mutator mice) induces premature aging and affects musculoskeletal tissues [24, 25].

The aim of this project was to evaluate whether the substitution of mtDNA would also result in changes in the joints during spontaneous aging. Our results showed that conplastic (BL/6^NZB^) mice developed fewer signs of aging than the original strain BL/6^C57^, demonstrating that the mtDNA haplotype can alter the aging response in joints, but without OA alterations.

In line with our results, very recently, Geurts’ group found that the accumulation of mtDNA mutations in a DNA mutator mouse model conferred a predisposition to elevated subchondral bone turnover and hypertrophy in calcified cartilage, but not to the accelerated development of osteoarthritis [26].

In this study, we first analyzed the influence of aging on the joint cartilage of both strains. To achieve this, we evaluated histopathological changes in the cartilage by a modified Mankin scoring system [16]. Three criteria, namely, structure, cellularity, and matrix staining of the cartilage, were analyzed and scored. In terms of the total Mankin scores, there were significant increases with the aging process among mice at 25, 75, and 90 weeks of age in BL/6^C57^ and conplastic (BL/6^NZB^) strains (Figure 1). Specifically, these differences at 25 and 75 weeks were significant for all three of the criteria analyzed. However, at 90 weeks of age, we observed a significant difference only in terms of the structure (data not shown).

Next, we continued comparing the Mankin scores of BL/6^C57^ and conplastic (BL/6^NZB^) mice. Interestingly, we found that conplastic (BL/6^NZB^) mice developed fewer signs of aging with a consequently lower Mankin score than the original strain BL/6^C57^ (Figure 1). We observed significant differences in all age groups analyzed. Specifically, the medial compartment and the medial femoral condyle were the joint regions where we found significant differences between conplastic (BL/6^NZB^) mice and the original strain BL/6^C57^ (Table 1), in accordance with the development of aging damage in human knee. Notably, we observed localized fibrillation and decreased chondrocyte density, but we did not find complete loss of cartilage typical of the OA process, which we observed in a model of surgically induced OA [27]. These findings on Mankin score levels reveal that mtDNA manipulation can attenuate the aging-related changes that affect the cartilage at joints, reducing their potential contribution to the development of OA. Besides, we analyzed the presence of histopathological changes in synovial tissue and subchondral bone (Supplementary Figure 2). Although we previously detected that conplastic (BL/6^NZB^) mice under a surgically-induced model of OA presented lower damage in these tissues than observed in an original strain BL/6^C57^ [27], in this study we failed to observed any significant difference between both strains in term of pathological changes in these articular tissues. It could be due to the fact that age-related spontaneous model of OA performed in this study is less aggressive and generally causes a lower articular damage. Nevertheless, conplastic (BL/6^NZB^) mice showed an attenuation in age-associated reduction of growth plate of tibial epiphysis in comparison with detected in BL/6^C57^ mice (Supplementary Figure 2).

Autophagy declines with aging and during OA [2]. Here, we measured two of the most important factors that participate in the autophagy process: LC3 and Beclin-1. The cartilage expression of LC3 and Beclin-1 was significantly increased in joints from conplastic (BL/6^NZB^) mice at 75 (Supplementary Figures 3) and 90 weeks of age (Figure 2) compared with those of BL/6^C57^ mice at the same ages. No significant differences were found in mice at 25 weeks of age (Supplementary Figures 3). Thus, our results confirmed that there were no basal differences between the two strains and that these differences appeared only in old age. Moreover, in our previous study using a model of surgically induced OA, we observed that conplastic (BL/6^NZB^) mice also presented more cartilage expression of LC3 and Beclin-1 than the original strain [27]. In order to confirm that increment of LC3 and Beclin-1 proteins reflected an augmentation of autophagy flux, we analyzed p62 levels, a classical receptor and indicator of autophagy activity as P62 accumulates when autophagy is inhibited, and decreased levels can be observed when autophagy is elicited [2]. Interestingly, BL/6^NZB^ mice showed lower levels of P62 than BL/6^C57^ mice, suggesting its higher degradation in the autolysosome and thus increased activation of autophagy flux in the conplastic mice. These findings imply that mtDNA variability affects the autophagic pathway in both aging and OA, suggesting a potential mechanism mediating the effects of mtDNA variants in the process of joint damage.

To study the senescence activity, we measured the cartilage expression of MMP13, β-Galactosidase, and p16, markers involved in the senescence process, and Ki67, an indicator of proliferation activity. Increased production of MMPs, in particular MMP13 and MMP3, was detected in cartilage with aging [28], while β-Galactosidase and p16 are widely used markers for cellular senescence [29–31]. In mice at 90 weeks of age, we found less cartilage expression of MMP13, β-Galactosidase, and p16 in joints from conplastic (BL/6^NZB^) mice than in the original strain, whereas proliferation activity measured as Ki67 expression was increased in BL/6^NZB^ strain (Figure 3). We did not detect significant differences in mice at 25 and 75 weeks of age (Supplementary Figure 4). These results are in accordance with our data on the surgically induced OA in the conplastic (BL/6^NZB^) mice and the original strain, where we did not observe differences between sham groups at 25 weeks of age [27]. Thus, at the basal time (25 weeks), there were no differences between the strains and only when aging progressed did we observe how different mtDNA variants influence the expression of senescence markers predisposing the mice to a worse disease outcome.

In chondrocytes, age-related mitochondrial dysfunction together with reduced activity of mitochondrial superoxide dismutase (SOD2) results in an increase in mitochondrially derived ROS with consequent DNA damage. Moreover, the aging-associated increase of MMP13 production in joint also contributes to the increase of ROS production [32]. To analyze oxidative stress, we measured the cartilage expression of 8-oxo-dG, an excellent biomarker, to determine the extent of oxidative damage. We found significantly less cartilage expression of 8-oxo-dG in joints from conplastic (BL/6^NZB^) mice at 90 weeks of age compared with that in BL/6^C57^ mice (Figure 4). Interestingly, we also found significant differences in the marker 8-oxo-dG in mice at 25 and 75 weeks of age (Supplementary Figure 5). Notably, in our recent study where we analyzed the effect of surgically induced OA in conplastic (BL/6^NZB^) mice compared with that in the original strain BL/6^C57^, we also observed a significant basal (sham group) difference in 8-oxo-dG between the two strains at 25 weeks of age. Thus, this marker of oxidative stress was less expressed in cartilage from sham conplastic (BL/6^NZB^) mice than in the sham original strain [27]. These findings suggest that mitochondrially derived oxidative stress could be the first potential mechanism for initiating the aging process, characterizing the different responses between the two strains, and contributing to the development of OA. Aging-associated decreases in autophagy and the accumulation of ROS and MMPs induce chondrocyte apoptosis with consequent cartilage damage [2]. Here, we measured the cartilage expression of cleaved-caspase 3 in both studied strains. BL/6^C57^ mice at 90 weeks of age showed more CC3 expression in cartilage than conplastic (BL/6^NZB^) mice (Figure 4). We did not find significant differences between the strains in mice at 25 and 75 weeks of age (Supplementary Figure 5).

Notably, we also observed differences in the autophagic and apoptotic responses, at the joint level, between conplastic (BL/6^NZB^) mice and the BL/6^C57^ strain, in a model after the surgical induction of OA. Thus, these mechanisms seem to mediate the effects of the mtDNA variants and be key factors in both processes. These findings further shed light on the pivotal role of harboring specific mtDNA haplogroup and cluster such as J or JT [12, 23] in the OA pathogenesis; mitochondrial polymorphisms that have also been associated, like conplastic (BL/6^NZB^) mice [15], to higher longevity and lower mtDNA damage and production of age-related mitochondrial ROS/RNS. Likewise, they could represent a potential novel approach to target aging-related changes in the joints in order to prevent or slow the development and progression of OA.

Nonetheless, the study has some limitations that must be highlighted. We here employed conplastic mice with NZB/OlaHsd mitochondrial genome and C57BL/6JOlaHsd nuclear DNA in order to evaluate the role of mitochondrial variants in OA development. Reciprocal conplastic animals, that is, animals with the NZB/OlaHsd nuclear genome and C57BL/6JOlaHsd mitochondrial DNA were not assayed as having lesser therapeutic significance, although it would be interesting to test with the aim of further confirming our current findings. In addition, activation of pathological pathways in the cartilage was evaluated by histological and immunohistochemical analysis by different markers for each process, however future studies should be warranted to reinforce our findings.

**Figure 5:**
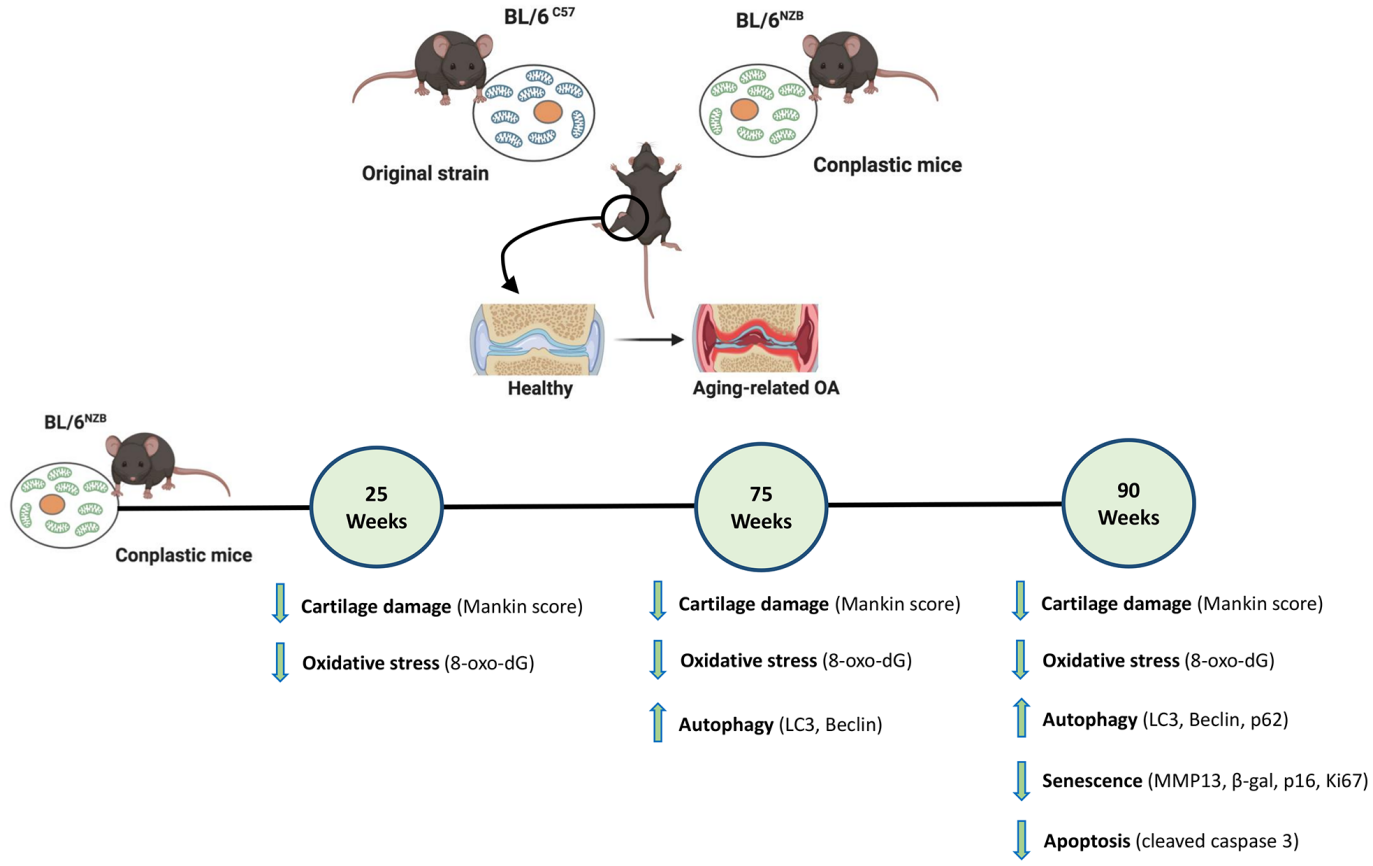
**Schematic representation of the joint aging model in conplastic (BL/6^NZB^) mice and the original strain BL/6^C57^.** 8-oxo-2′-deoxyguanosine (8-oxo-dG); Cleaved caspase-3 (CC3); Matrix metalloproteinase 13 (MMP13). Created with BioRender.com.

In conclusion, our findings demonstrated that, in response to aging, conplastic (BL/6^NZB^) mice showed reduced joint deterioration compared with the original strain BL/6^C57^. Moreover, we found that mtDNA variants differentially modulate the aging process at the joint level through modulating autophagy, senescence, oxidative stress, and apoptosis (Figure 5). Taken together, our results support the hypothesis that the mtDNA background plays a role in the process of joint damage, confirming that mtDNA variability is a prognostic factor for OA in aging and suggesting that modulation of mitochondrial oxidative phosphorylation (OXPHOS) is a novel therapeutic target in OA associated with aging.

## MATERIALS AND METHODS

### Animals

The conplastic mouse strain was generated in the laboratory of Dr. José Antonio Enríquez [17] by backcrossing females (mitochondrial donors) with males of the parental recipient strain. Parental C57BL/6JOlaHsd and NZB/OlaHsd strains were purchased from Harlan Laboratories. Specifically, the conplastic (BL/6^NZB^) mouse strain was developed with the C57BL/6JOlaHsd nuclear genome and NZB/OlaHsd mtDNA for comparison with the original C57BL/6JOlaHsd strain (BL/6^C57^). Mice were bred at the Experimental Surgery Unit of A Coruña under standard conditions (12:12 light:dark cycle, 22 ± 1° C temperature, 50%–60% humidity), and food and water were provided *ad libitum*. After the experiments, the animals were sacrificed using carbon monoxide, followed by cervical dislocation.

Animal experiments were carried out in accordance with the legislation for the protection of animals (European Directive 2010/63) used for scientific purposes and in compliance with the ARRIVE guidelines. The study was approved by the local ethical committee of animal experimentation “Comité de Ètica de Experimentación Animal de la Xerencia de Xestión Integrada A Coruña” (CEEA-XXIAC) and by the “Consellería do Medio Rural” of Xunta de Galicia (15002/2018/01).

### Aging model

Conplastic (BL/6^NZB^) mice and the original C57BL/6JOlaHsd strain (BL/6^C57^) were used as aging models. For each strain, the mice were divided into three age groups (all females) corresponding to 25 (BL/6^C57^= 5; conplastic (BL/6^NZB^) n=5), 75 (BL/6^C57^= 6; conplastic (BL/6^NZB^) n=6), and 90 (BL/6^C57^= 6; conplastic (BL/6^NZB^) n=7) weeks old. The mice were kept under normal conditions (free movement, without any mechanical or physical stress) and sacrificed at 25, 75, and 90 weeks of age.

### Histological analysis

Knee joints from the mice were harvested, fixed overnight in 3.7%–4% formaldehyde, decalcified in Decal (HistoLab) for 6 h at 100 rpm, and then subjected to paraffin embedding. Serial sections (4 μm) were cut, deparaffinized in xylene, and then subjected to a graded series of alcohol washes.

Left and right hind knee joints from the conplastic (BL/6^NZB^) mice as well as from the BL/6^C57^ mice at 25, 75, and 90 weeks of age were processed and cut into coronal sections for histological analysis. All sections were subjected to hematoxylin-eosin and Safranin O-fast green staining and histopathology graded using a modified Mankin scoring system [16]. All quadrants of the joint (medial tibial plateau, medial femoral condyle, lateral tibial plateau, and lateral femoral condyle) were scored separately and averaged (Supplementary Figure 1). Three criteria were selected for histological assessment of each quadrant: structure, cellularity, and matrix staining of the cartilage. Sections were analyzed in a blinded manner and scored by two different experienced scientists (MS and CVG). Average scores were used in the statistical analyses.

### Immunohistochemistry

Mouse knee joint sections were deparaffinized, washed, and incubated with primary antibody to MMP13 (ab 39012; Abcam; 1:100), LC3 (PM036; MBL; 1:1000), Beclin-1 (ab 62557; Abcam; 1:200), or cleaved caspase-3 (#9664; Cell Signaling; 1:100) for 1 h at room temperature, or Ki67 (ab 15580; Abcam; 1:400), P62 (MAB8028;R&D Systems; 1:200), p16 (ab54210; Abcam; 1:200) overnight at 4° C, followed by use of the Dako REAL™ EnVision™ Detection System, Peroxidase/DAB+ (K-5007, Dako). For detection of the antibody to 8-oxo-dG (ab 48508; Abcam; 1:180), Animal Research Kit (ARK™) Peroxidase was used. To detect β-Galactosidase (ab 9361; Abcam; 1:200), the secondary antibody Goat Anti-Chicken IgY H&L (HRP) (ab 6877; Abcam; 1:1000) was added for 1 h at room temperature. Finally, the slides were mounted with DePEX mounting medium (Leica Biosystems).

For both right and left mouse knee joints from the aging models, three images representing the medial tibial plateau and medial femoral condyle were taken using a microscope (LEICA ICC50W). The total number of chondrocytes and the number of positive cells for the corresponding antibody were counted in the microscopic field in each section [33]. The values are expressed as the proportion of positive cells relative to the total number of chondrocytes in the joint medial compartment.

### Statistical analysis

The results are given as mean ± standard error of the mean (SEM) and statistical analysis was performed using the non-parametric Mann-Whitney test. *p* values less than 0.05 were considered significant. Data were analyzed using the Prism computerized package (GraphPad Prism Software v. 6.0, San Diego, CA, USA).

## Supplementary Material

Supplementary Figures
