# Supplementary material for: mtDNA variability determines spontaneous joint aging damage in a conplastic mouse model
Source: Aging (Albany NY). 2022 Jul 2;14(15):5966–83. doi: 10.18632/aging.204153 (PMC9417242; doi:10.18632/aging.204153)
Supplement: Supplementary Figures [file aging-14-204153-s001.pdf]

SUPPLEMENTARY FIGURES

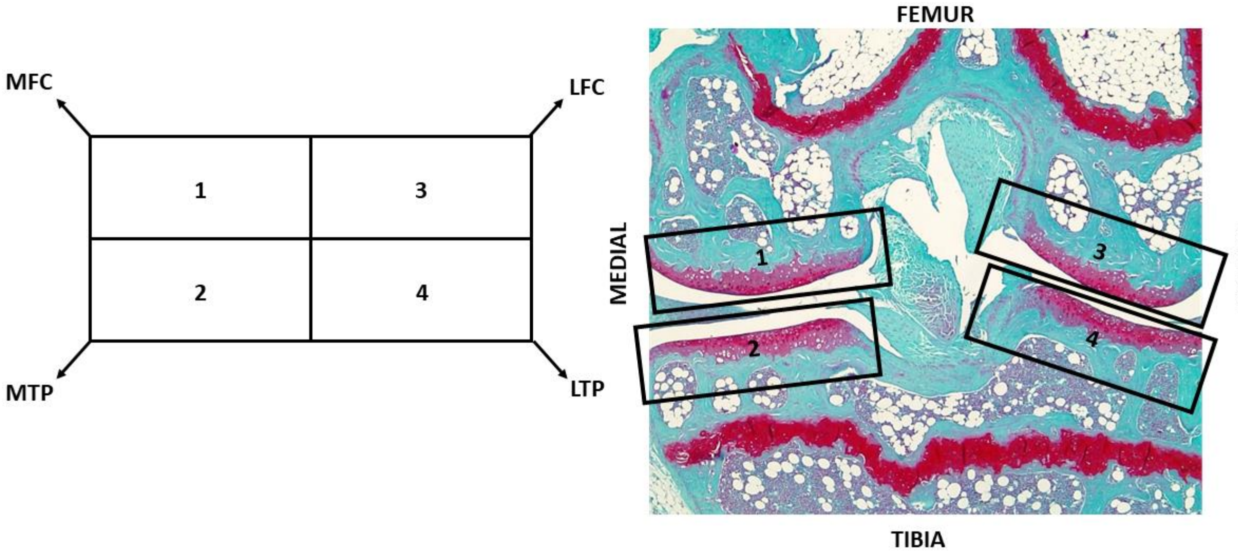

Supplementary Figure 1. Coronal section analysis for Mankin score measurement. TP (tibial plateau) = MTP+LTP; FC (femoral condyle) = MTP+LFC; medial compartment= MTP+MFC; lateral compartment = LTP+LFC; whole joint: medial compartment + lateral compartment.

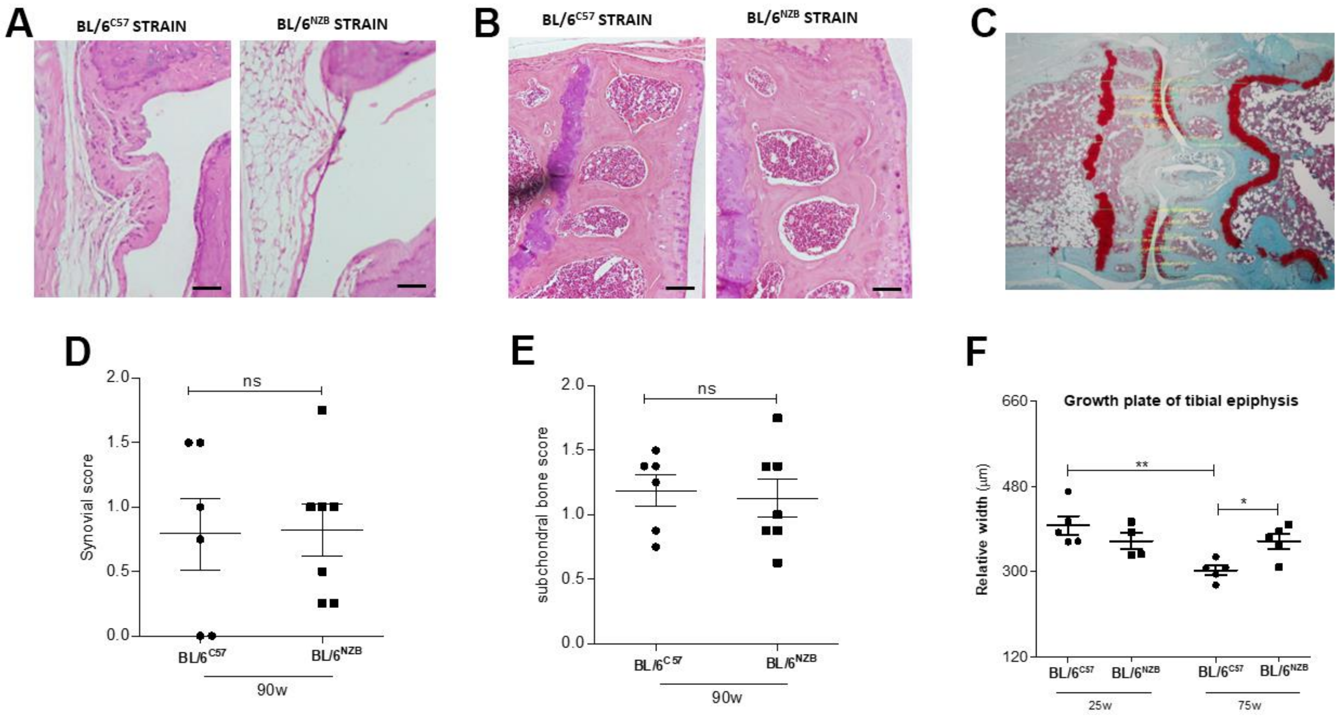

Supplementary Figure 2. Histopathological evaluation of synovial tissue and subchondral bone and histomorphometric analysis of growth plate of tibial epiphysis. Representative images of synovial tissue (**A**) and subchondral bone (**B**) from joint sections of BL/6^{C57} and conplastic (BL/6^{NZB}) mice at 90 weeks of age stained with Hematoxylin-Eosin. Histopathological changes in the synovium (**D**) and subchondral bone (**E**) were graded by semi-quantitative scoring system (https://doi.org/10.1111/j.1365-2559.2006.02508.x; https://doi.org/10.1371/journal.pone.0173726; respectively). Original magnification: 20×. Scale bar, 100 µm. Graphs represent means ± SEM; n=6 in BL/6^{C57} and n=7 in conplastic (BL/6^{NZB}) mice. (**C**, **F**) Histomorphometric analysis of growth plate of tibial epiphysis from joint sections of BL/6^{C57} and conplastic (BL/6^{NZB}) mice at 25 and 75 weeks. Representative image of analysis performed with Software NIS Elements associated to the microscope NIKON (BX61). Graphs represent means ± SEM; n=5 in BL/6^{C57} and n=4 in conplastic (BL/6^{NZB}) mice at 25 weeks; n=5 in BL/6^{C57} and conplastic (BL/6^{NZB}) mice at 75 weeks. ns=not significant, *p<0.05, **p<0.01 by non-parametric Mann-Whitney test.

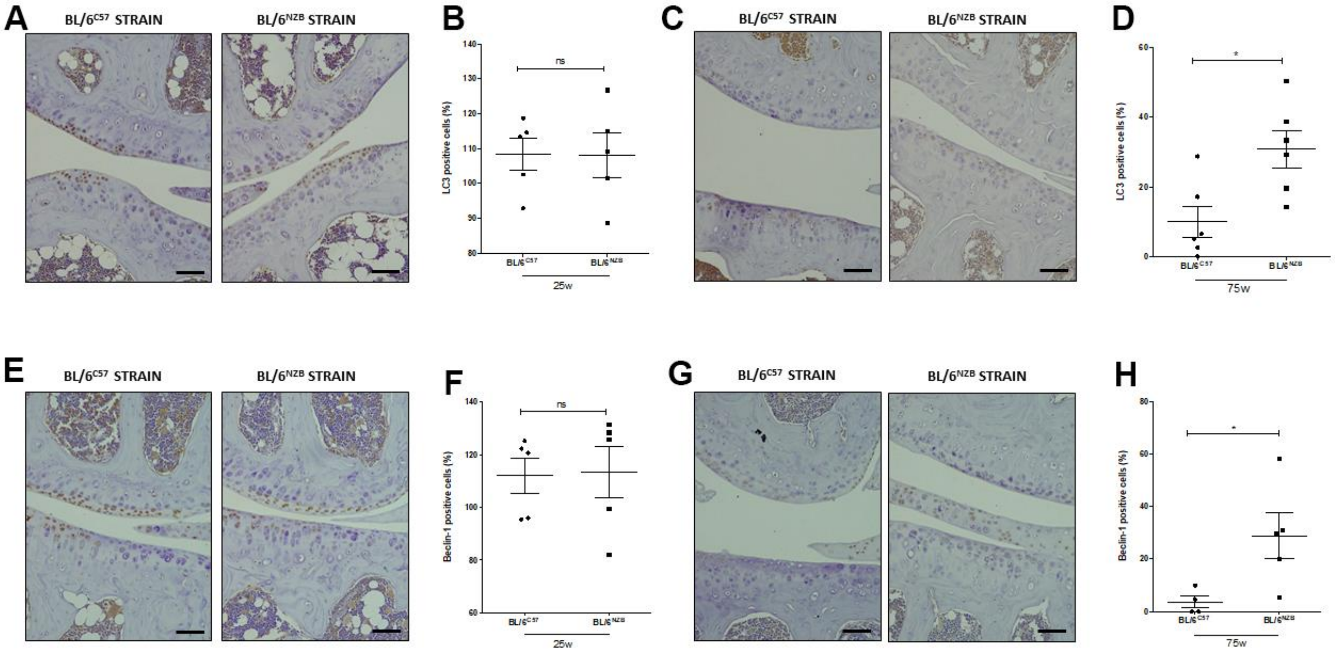

Supplementary Figure 3. LC3 and Beclin-1 expression in cartilage from mice at 25 and 75 weeks of age. Representative images of medial compartment of knee joints from $BL/6^{C57}$ and conplastic ($BL/6^{NZB}$) mice at 25 weeks (**A**) and 75 weeks (**C**) of age stained with LC3. Quantitative analysis of LC3-positive cells of knee joints from $BL/6^{C57}$ and conplastic ($BL/6^{NZB}$) mice at 25 weeks (**B**) and 75 weeks (**D**) of age. 25 weeks: n=5 in $BL/6^{C57}$ and n=5 conplastic ($BL/6^{NZB}$) mice; 75 weeks: n=6 in $BL/6^{C57}$ and n=6 conplastic ($BL/6^{NZB}$) mice. Representative images of medial compartment of knee joints from $BL/6^{C57}$ and conplastic ($BL/6^{NZB}$) mice at 25 weeks (**E**) 75 weeks (**G**) of age stained with Beclin-1. Quantitative analysis of Beclin-1-positive cells of knee joints from $BL/6^{C57}$ and conplastic ($BL/6^{NZB}$) mice at 25 weeks (**F**) 75 weeks (**H**) of age. 25 weeks: n=5 in $BL/6^{C57}$ and n=5 conplastic ($BL/6^{NZB}$) mice; 75 weeks: n=4 in $BL/6^{C57}$ and n=5 conplastic ($BL/6^{NZB}$) mice. Original magnification: 20×. Scale bar, 50 µm. Graphs represent means ± SEM. ns=not significant, *p<0.05 by non-parametric Mann-Whitney test.

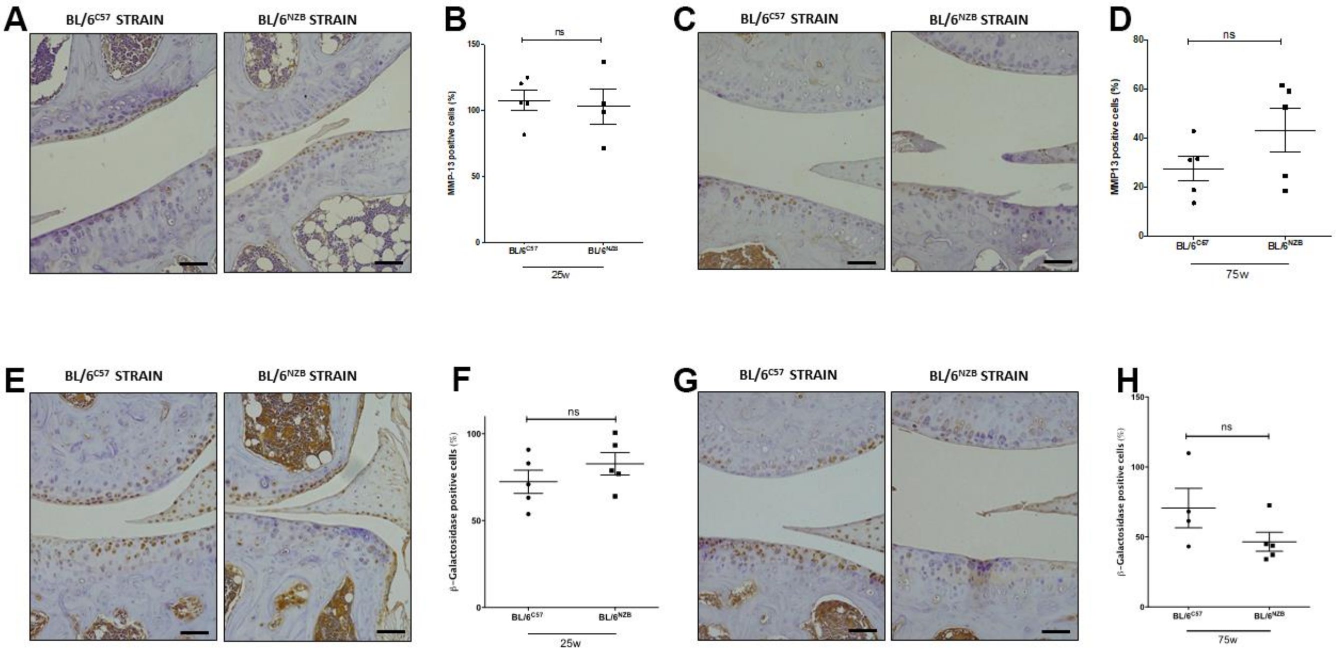

Supplementary Figure 4. Immunohistochemistry for MMP13 and β-Galactosidase in mice at 25 and 75 weeks. Representative images of medial compartment of knee joints from BL/6^{C57} and conplastic (BL/6^{NZB}) mice at 25 weeks (**A**) and 75 weeks (**C**) of age stained with MMP13. Quantitative analysis of MMP13-positive cells of knee joints from BL/6^{C57} and conplastic (BL/6^{NZB}) mice at 25 weeks (**B**) and 75 weeks (**D**) of age. 25 weeks: n=5 in BL/6^{C57} and n=4 conplastic (BL/6^{NZB}) mice; 75 weeks: n=5 in BL/6^{C57} and n=5 conplastic (BL/6^{NZB}) mice. Representative images of medial compartment of knee joints from BL/6^{C57} and conplastic (BL/6^{NZB}) mice at 25 weeks (**E**) and 75 weeks (**G**) of age stained with β-Galactosidase. Quantitative analysis of β-Galactosidase-positive cells of knee joints from BL/6^{C57} and conplastic (BL/6^{NZB}) mice at 25 weeks (**F**) and 75 weeks (**H**) of age. 25 weeks: n=5 in BL/6^{C57} and n=5 conplastic (BL/6^{NZB}) mice; 75 weeks: n=4 in BL/6^{C57} and n=5 conplastic (BL/6^{NZB}) mice. Original magnification: 20×. Scale bar, 50 µm. Graphs represent means ± SEM; ns=not significant by non-parametric Mann-Whitney test.

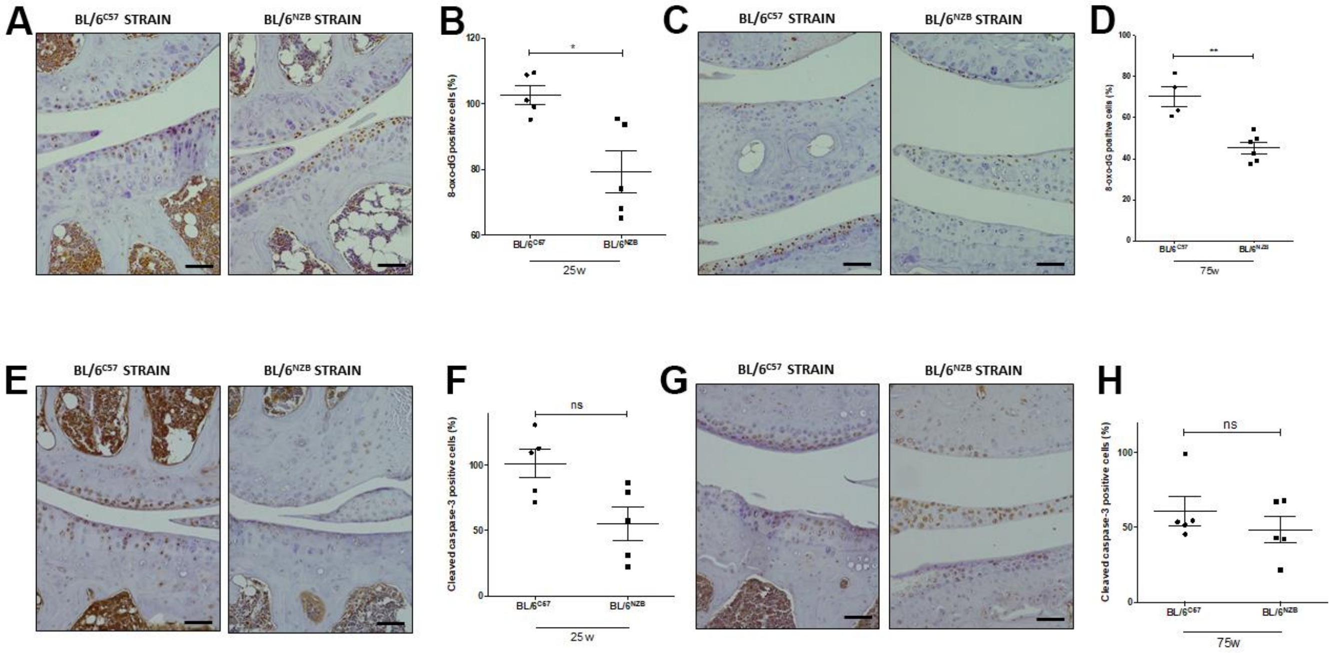

Supplementary Figure 5. 8-oxo-dG cartilage and cleaved caspase-3 expression in conplastic mice at 25 and 75 weeks. Representative images of medial compartment of knee joints from BL/6^{C57} and conplastic (BL/6^{NZB}) mice at 25 weeks (**A**) and 75 weeks (**C**) of age stained with 8-oxo-dG. Quantitative analysis of 8-oxo-dG-positive cells of knee joints from BL/6^{C57} and conplastic (BL/6^{NZB}) mice at 25 weeks (**B**) and 75 weeks (**D**) of age. 25 weeks: n=5 in BL/6^{C57} and n=5 conplastic (BL/6^{NZB}) mice; 75 weeks: n=4 in BL/6^{C57} and n=6 conplastic (BL/6^{NZB}) mice. Representative images of medial compartment of knee joints from BL/6^{C57} and conplastic (BL/6^{NZB}) mice at 25 week (**E**) 75 weeks (**G**) of age stained with cleaved caspase-3. Quantitative analysis of cleaved caspase-3 -positive cells of knee joints from BL/6^{C57} and conplastic (BL/6^{NZB}) mice at 25 weeks (**F**) and 75 weeks of age (**H**). 25 weeks: n=5 in BL/6^{C57} and n=5 conplastic (BL/6^{NZB}) mice, 75 weeks: n=5 in BL/6^{C57} and n=5 conplastic (BL/6^{NZB}) mice. Original magnification: 20×. Scale bar, 50 µm. Graphs represent means ± SEM; ns=not significant, *$p<0.05$, **$p<0.01$ by non-parametric Mann-Whitney test.
